# Assessment of a COVID-19 Control Plan on an Urban University Campus During a Second Wave of the Pandemic

**DOI:** 10.1001/jamanetworkopen.2021.16425

**Published:** 2021-06-25

**Authors:** Davidson H. Hamer, Laura F. White, Helen E. Jenkins, Christopher J. Gill, Hannah E. Landsberg, Catherine Klapperich, Katia Bulekova, Judy Platt, Linette Decarie, Wayne Gilmore, Megan Pilkington, Trevor L. MacDowell, Mark A. Faria, Douglas Densmore, Lena Landaverde, Wenrui Li, Tom Rose, Stephen P. Burgay, Candice Miller, Lynn Doucette-Stamm, Kelly Lockard, Kenneth Elmore, Tracy Schroeder, Ann M. Zaia, Eric D. Kolaczyk, Gloria Waters, Robert A. Brown

**Affiliations:** 1 Department of Global Health, Boston University School of Public Health, Boston, Massachusetts; 2 Section of Infectious Disease, Department of Medicine, Boston University School of Medicine, Boston, Massachusetts; 3 National Emerging Infectious Disease Laboratory, Boston, Massachusetts; 4 Precision Diagnostics Center, Boston University, Boston, Massachusetts; 5 Department of Biostatistics, Boston University School of Public Health, Boston, Massachusetts; 6 Student Health Services, Healthway, Boston University, Boston, Massachusetts; 7 Department of Biomedical Engineering, Boston University, Boston, Massachusetts; 8 Information Services and Technology, Boston University, Boston, Massachusetts; 9 Boston University Analytical Services & Institutional Research, Boston, Massachusetts; 10 Electrical and Computer Engineering, Boston University, Boston, Massachusetts; 11 Biological Design Center, Boston University, Boston, Massachusetts; 12 Department of Mathematics and Statistics, Boston University, Boston, Massachusetts; 13 Human Resources, Boston University, Boston, Massachusetts; 14 Office of External Affairs, Boston University, Boston, Massachusetts; 15 BU Clinical Testing Laboratory, Research Department, Boston University, Boston, Massachusetts; 16 Continuous Improvement & Data Analytics, Boston University, Boston, Massachusetts; 17 Office of the Provost, Boston University, Boston, Massachusetts; 18 Occupational Health Center, Boston University, Boston Massachusetts; 19 Hariri Institute for Computing, Boston University, Boston, Massachusetts; 20 College of Health and Rehabilitation Services, Sargent College, Boston University, Boston, Massachusetts; 21 College of Engineering, Boston University, Boston, Massachusetts; 22 Office of the President, Boston University, Boston, Massachusetts

## Abstract

**Importance:**

The COVID-19 pandemic has severely disrupted US educational institutions. Given potential adverse financial and psychosocial effects of campus closures, many institutions developed strategies to reopen campuses in the fall 2020 semester despite the ongoing threat of COVID-19. However, many institutions opted to have limited campus reopening to minimize potential risk of spread of SARS-CoV-2.

**Objective:**

To analyze how Boston University (BU) fully reopened its campus in the fall of 2020 and controlled COVID-19 transmission despite worsening transmission in Boston, Massachusetts.

**Design, Setting, and Participants:**

This multifaceted intervention case series was conducted at a large urban university campus in Boston, Massachusetts, during the fall 2020 semester. The BU response included a high-throughput SARS-CoV-2 polymerase chain reaction testing facility with capacity to deliver results in less than 24 hours; routine asymptomatic screening for COVID-19; daily health attestations; adherence monitoring and feedback; robust contact tracing, quarantine, and isolation in on-campus facilities; face mask use; enhanced hand hygiene; social distancing recommendations; dedensification of classrooms and public places; and enhancement of all building air systems. Data were analyzed from December 20, 2020, to January 31, 2021.

**Main Outcomes and Measures:**

SARS-CoV-2 diagnosis confirmed by reverse transcription–polymerase chain reaction of anterior nares specimens and sources of transmission, as determined through contact tracing.

**Results:**

Between August and December 2020, BU conducted more than 500 000 COVID-19 tests and identified 719 individuals with COVID-19, including 496 students (69.0%), 11 faculty (1.5%), and 212 staff (29.5%). Overall, 718 individuals, or 1.8% of the BU community, had test results positive for SARS-CoV-2. Of 837 close contacts traced, 86 individuals (10.3%) had test results positive for COVID-19. BU contact tracers identified a source of transmission for 370 individuals (51.5%), with 206 individuals (55.7%) identifying a non-BU source. Among 5 faculty and 84 staff with SARS-CoV-2 with a known source of infection, most reported a transmission source outside of BU (all 5 faculty members [100%] and 67 staff members [79.8%]). A BU source was identified by 108 of 183 undergraduate students with SARS-CoV-2 (59.0%) and 39 of 98 graduate students with SARS-CoV-2 (39.8%); notably, no transmission was traced to a classroom setting.

**Conclusions and Relevance:**

In this case series of COVID-19 transmission, BU used a coordinated strategy of testing, contact tracing, isolation, and quarantine, with robust management and oversight, to control COVID-19 transmission in an urban university setting.

## Key Points

**Question:**

Can a multifaceted approach lead to control of COVID-19 transmission and spread on an urban campus?

**Findings:**

In this case series including more than 500 000 COVID-19 tests and 719 individuals with COVID-19 at Boston University, active surveillance of campus populations, isolation of individuals with SARS-CoV-2 infection, early and vigorous contact tracing and quarantine, regular communication, robust data systems, and strong leadership were associated with minimal transmission of SARS-CoV-2. Most transmission occurred off campus, and there was no evidence of classroom transmission.

**Meaning:**

These findings suggest that using frequent testing, vigorous contact tracing, rapid isolation and quarantine, and a strong leadership structure to ensure rapid decision-making and adaption to emerging data, controlling the spread of SARS-CoV-2 on an urban campus was feasible despite worsening local transmission during the semester.

## Teaser

This case series describes the use of a multifaceted COVID-19 control plan to reduce spread of SARS-CoV-2 at a large urban university during the second wave of the pandemic.

## Introduction

The SARS-CoV-2 global pandemic resulted in nearly 1.8 million deaths worldwide in 2020.^1^ The initial surge of US COVID-19 cases had a devastating impact on universities and colleges owing to widespread campus closures in spring 2020.^2^ Faced with serious financial challenges and adverse social impacts associated with continued closure, some universities developed multilayered COVID-19 risk mitigation strategies to allow campuses to reopen during the fall 2020 semester.^3,4^

Boston University (BU) is a private university with a student, staff, and faculty population of approximately 40 000 individuals located in the heart of a large US city (Boston, Massachusetts), a scenario for potential widespread COVID-19 transmission. Despite these challenges, the BU administration pursued an aggressive risk mitigation strategy involving widespread asymptomatic screening for COVID-19, environmental modifications, classroom dedensification, contact tracing, isolation, and quarantine to allow its students to return to in-person learning in the fall 2020 semester. Here, we describe the BU experience as a case series offering important lessons that may be broadly applicable to other higher education institutions.

## Methods

The plan for this case series was reviewed by the BU Charles River Campus (CRC) institutional review board and was classified as non–human participants research and therefore exempt for approval and informed consent. The BU Medical Campus (BUMC) institutional review board reviewed the safe behavior quality improvement project (eAppendix in the Supplement) and determined it to be exempt from review and informed consent.

**Figure 1.**
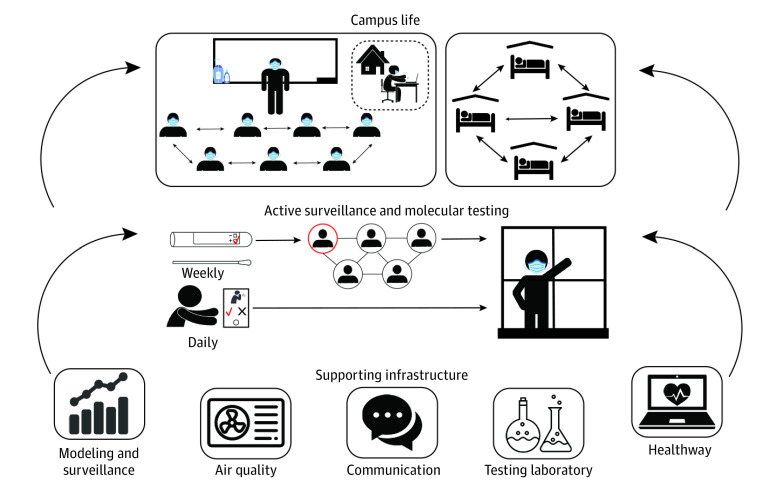
Schematic of the Major Components of the Campus COVID-19 Control Strategies A multifaceted approach was used for COVID-19 control on the Boston University campus, using modeling; active surveillance with molecular testing for SARS-CoV-2 of all students, faculty, and staff on campus; daily health attestations; dedensification of classrooms; and optimizing air quality and filtration in buildings. This complex strategy required an integrated tracking system (Healthway) and effective communication to the whole Boston University community.

Initial planning, starting in March 2020, centered on active surveillance for individuals with asymptomatic and symptomatic SARS-CoV-2 infections via on-site molecular testing (ie, polymerase chain reaction testing [PCR]) for SARS-CoV-2 (eAppendix in the Supplement). This involved setting up systems for high-throughput laboratory testing, timely communication of results, rapid contact tracing, isolation of infected individuals, and quarantine of close contacts (Figure 1).

Surveillance was complemented by additional control measures, including mask use, enhanced hygiene practices, social distancing recommendations, daily health attestations, dedensification of classrooms and public places, and enhancement of all building air systems. This process was aided by mathematical modeling, multiple data systems, 24 hours per day, 7 days per week monitoring, and data-driven strategy refinements throughout the semester. This required a coordinated leadership and management structure (eFigure 1 in the Supplement), through interlocking management committees and leadership groups and communication strategies.

### Setting

BU, a large urban university in Boston, Massachusetts, has 2 campuses: the larger main campus (CRC) with more than 30 000 members, and a smaller medical campus (BUMC, consisting of the Schools of Dentistry, Graduate Medical Sciences, Medicine, and Public Health) with nearly 10 000 members. In fall 2019, there were 11 439 students living on campus, 23 150 students living off campus, 10 514 staff members, and 4169 faculty members (faculty and staff numbers exclude overseas operations).

### Management

Starting in spring 2020, several groups were convened to coordinate COVID-19 control efforts on campus, including monitoring incoming data, modifying campus operations, implementing best public health and medical practices, surveillance, and budgeting (eAppendix and eFigure 1 in the Supplement). Central leadership at BU used the best available scientific evidence and guidance from national, state, and local authorities to develop policies and protocols for the systemic integration of students and employees back to the physical campus and for control of COVID-19 transmission through contact tracing, quarantine, and isolation.

### Learn From Anywhere

BU implemented hybrid teaching in which all undergraduate and graduate students could attend classes in-person or remotely. This allowed classes to simultaneously accommodate online-only students using video conference software (Zoom Video Communications) and students receiving instruction in person in classrooms.^5^ Workplace adjustments, including an option for online-only teaching, were granted for those at high risk for contracting COVID-19, such as older faculty members or those with high-risk medical conditions.

### BU SARS-CoV-2 Testing

#### Testing Laboratory

BU developed its own PCR testing laboratory. Test site staff observed anterior nasal swab sample self-collection by students, faculty, and staff (eAppendix in the Supplement).

#### Information Technology

A system was developed by BU’s Information Services and Technology experts linking electronic medical record systems for students, faculty, and staff and the laboratory information system in the testing facility with the web-based system for daily symptom attestation of all community members on campus, reservations for tests, and negative test reporting. This system was also used to track adherence of students, faculty, and staff for the routine testing and daily symptom attestation requirements.

#### SARS-CoV-2 Testing Categories

Based on guidance from public health authorities, BU developed 4 SARS-CoV-2 testing categories, reflecting different risks of exposure. These categories determined testing frequency, ranging from twice weekly for category 1 (eg, on-campus undergraduates) to no testing for category 4 (eg, students, faculty, or staff entirely off-campus) (eAppendix in the Supplement).

#### Managing and Responding to Test Results

All individuals with a negative test result were automatically notified by email to access their test results through a secure website. University health staff directly contacted individuals with positive test results.

### Contact Tracing

The contact tracing protocol was based on Centers for Disease Control and Prevention (CDC) and Massachusetts Community Tracing Collaborative processes,^6,7^ with adaptation from BU academic programs and student input. Contact tracers followed a detailed script to identify all close contacts, which were defined as someone within 6 feet of a person with SARS-CoV-2 infection for 15 minutes or more over a 24-hour period (eAppendix in the Supplement).

### Isolation, Quarantine, and Additional Campus Control Measures

Students with test results positive for SARS-CoV-2 had to isolate for 10 days after symptom onset and resolution of fever for at least 24 hours, and with improvement of other symptoms, or for 10 days from the positive test date if asymptomatic.^8^ Students identified as close contacts had to quarantine for 14 days from the exposure date. Based on evolving data from the CDC, this period was reduced to 10 days of quarantine on November 20, 2020.^9^ Students living on campus with confirmed SARS-CoV-2 were moved to special quarantine dormitories (maximum capacity, 650 units), and those living on campus who were determined to have been in close contact with infected students were moved to isolation dormitories (maximum capacity, 342 units) (eAppendix in the Supplement). Additional measures, such as face mask use, enhanced hand hygiene, social distancing recommendations, daily health attestations, dedensification of classrooms and public places, and enhancement of all building air systems, including optimization of filtration units, were implemented (eAppendix in the Supplement).

### Mathematical Modeling

We used probabilistic susceptible-exposed-infectious-recovered (SEIR) transmission modeling during summer 2020 to understand the expected relative efficacy of interventions for reducing COVID-19 transmission in the BU community to achieve only linear increases of cases from transmission outside BU (eAppendix in the Supplement). We used a stochastic agent-based model, implemented using the COVID-19 agent-based simulator (covasim) framework.^10^

### Communications, Surveillance, and Data Management

BU developed a dedicated COVID-19 external communications platform called *Back2BU*.^11^ The preexisting ecosystem of data warehousing and analysis systems was augmented to support the data storage, management, and analysis requirements necessary to allow for near real-time surveillance of BU’s COVID-19 response. Surveillance efforts focused on monitoring not only standard metrics around incidence, isolation, and quarantine, but also testing, contact tracing, and adherence with campus control measures. An external dashboard was created and updated daily to allow anyone to track BU metrics (eAppendix in the Supplement). In addition, an augmented internal dashboard was created to aid the various groups working with BU leadership to monitor and adapt the BU COVID-19 response.

### Adherence

Adherence with testing and attestation of symptoms were tracked electronically from October. The dean of students collected reports of violations of campus mandates, including gatherings, masking, and physical distancing, and Human Resources monitored faculty and staff adherence (eAppendix in the Supplement). Spot checks of adherence were conducted in parallel using trained observers (eAppendix in the Supplement).

### Fall 2020 Move-In

A comprehensive staggered return to campus schedule was adopted to reduce crowding and lines. The staggered return schedule provided time for students to be tested and for the University to respond to any positive results on arrival (eAppendix in the Supplement).

### Statistical Analysis

We describe the fall 2020 experience descriptively and report statistics (means, medians, and SDs) as appropriate. No statistical tests were performed. Data were analyzed from December 20, 2020, to January 31, 2021.

## Results

### Operational Results

#### Housing

While most graduate students (7266 students [98.6%]) lived off campus, most undergraduates lived in on-campus housing, including a total of 7131 students as of October 13, 2020, representing 67.6% of the fall 2020 capacity. Owing to dedensification efforts, 3453 students (48.4%) lived alone and 3678 students (51.6%) lived with 1 roommate (eAppendix in the Supplement).

#### Testing

**Figure 2.**
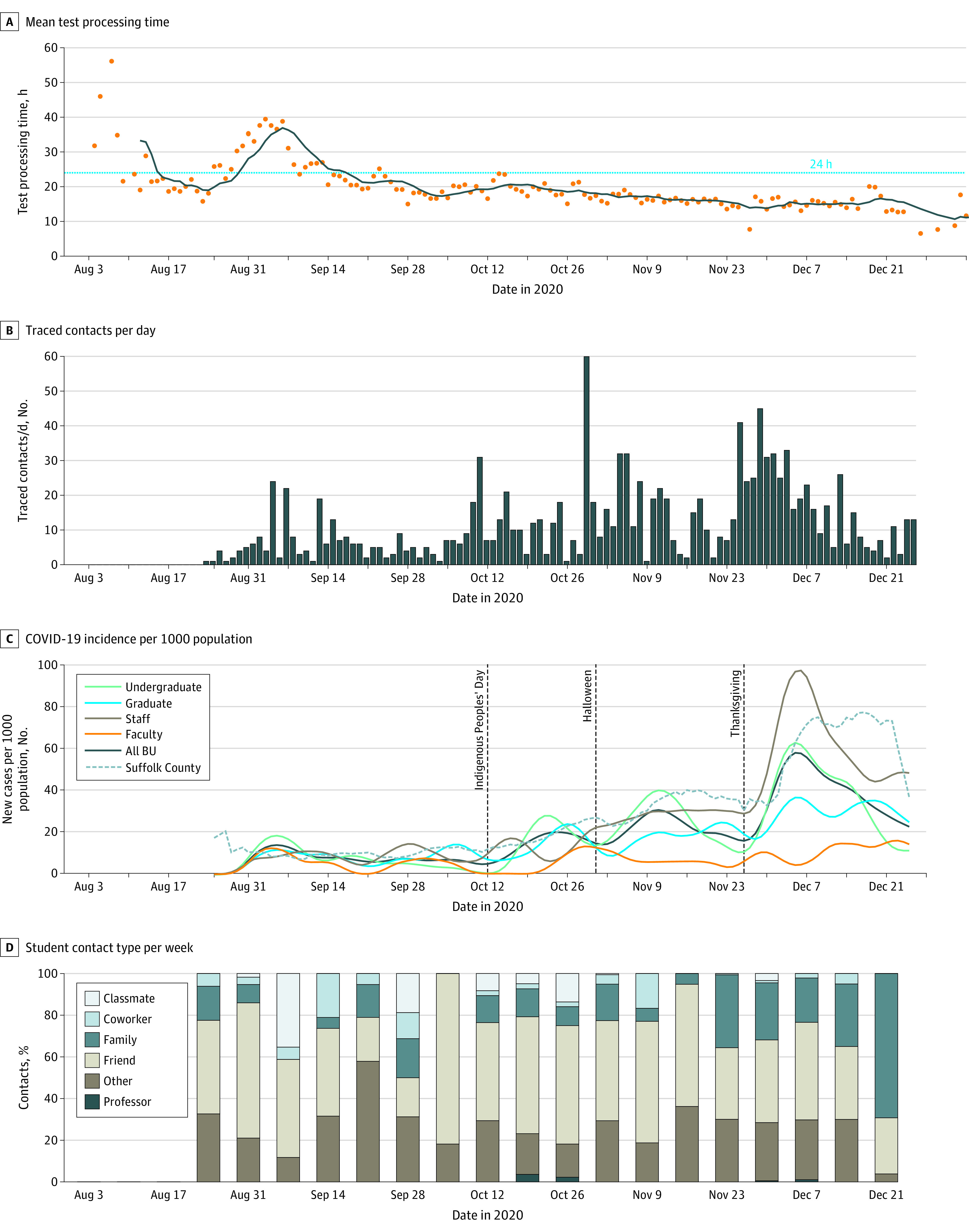
Synopsis of COVID at Boston University (BU) During the Fall 2020 Semester

Initial operations, staffing, and supplies aimed for a maximum of 6200 tests per day (approximately 42 000 tests per week) and to turn around results within 24 hours of collection. The laboratory reached a stable run rate by October, with a mean (SD) of 4543 (906.4) tests per day, with lower volume on weekends. Testing turnaround times decreased dramatically over the semester, leveling off to approximately 12 to 15 hours between sample collection to receipt of results (Figure 2A). The BU testing laboratory conducted 467 382 tests during the fall semester (517 357 tests including presemester move-in tests).

#### Contact Tracing, Quarantine, and Isolation

Within a mean (SD) 6.4 (6.4) hours (median [range], 3.0 [0-42] hours) after receipt of a positive test results, close contacts of an individual with a positive result for SARS-CoV-2 infection were notified of their exposure. However, this overall mean conceals improvements over time, decreasing from a mean (SD) of 10.1 (2.8) hours in September to 5.4 (6.3) hours in December 2020. Students had a mean (SD) of 3.18 (3.07) close contacts, and employees (faculty and staff) identified 0.82 (1.62) close contacts each. The number of individuals needing to be traced increased with increasing case numbers throughout the semester (Figure 2B). Despite having quarantine capacity for 650 individuals and isolation capacity for 342 individuals, occupancy only reached a maximum of 13.7% for quarantine and 12.9% for isolation at any one time (eFigure 3 in the Supplement).

#### Adherence

**Figure 3.**
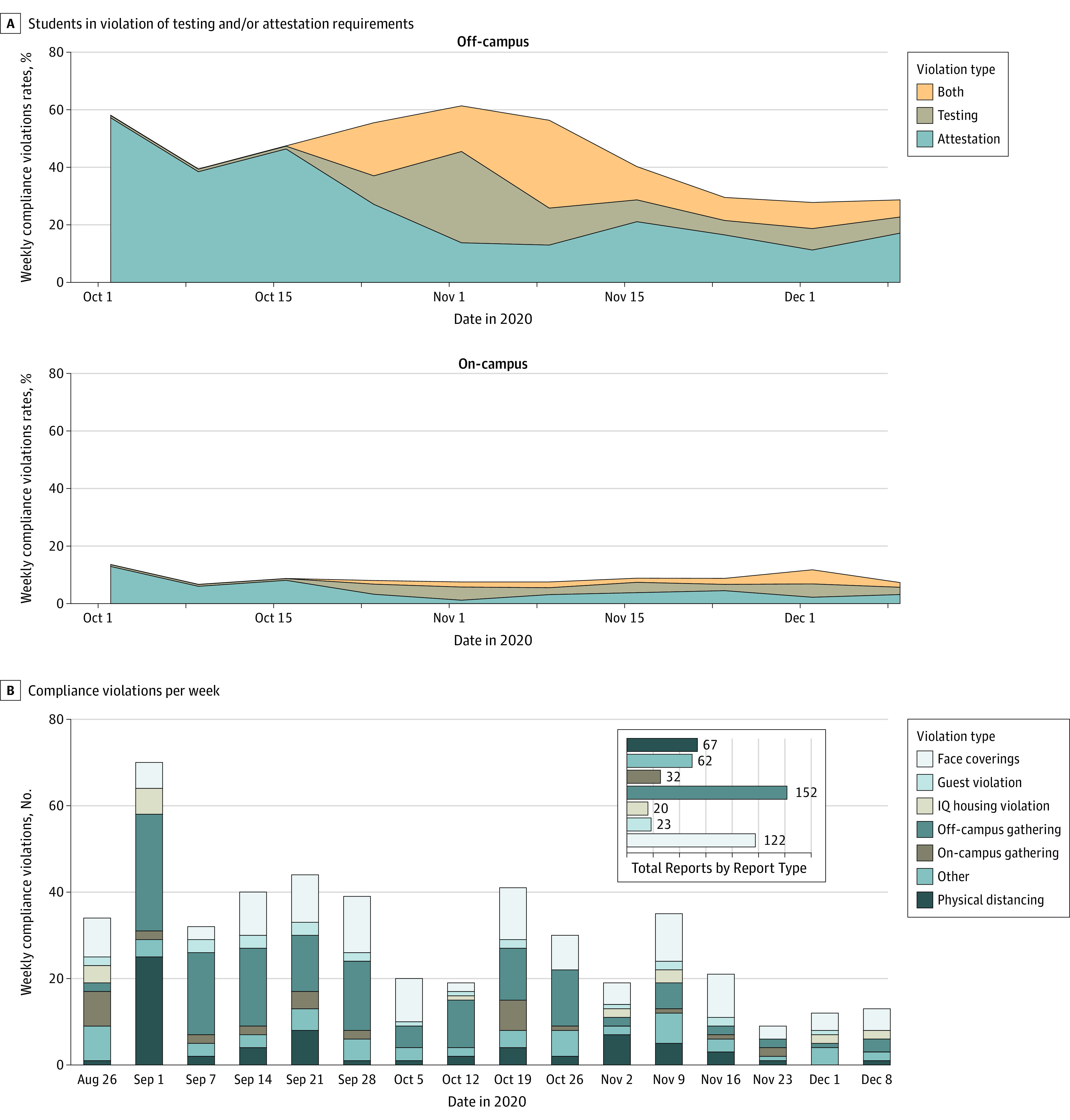
Statistics on Adherence to Testing or Attestation Requirements IQ indicates isolation/quarantine.

A mean (SD) of 583 (251) on-campus students (8%) per month were nonadherent with testing or attestation protocols in October and November, less than off-campus students during the same time (mean (SD), 4763.7 [1204] students [44.9%]) for off-campus students in categories 1, 2, and 3 (Figure 3A). Reported violations were most common as the semester began, but gradually tapered off (Figure 3B). Early semester violations were largely off-campus gatherings, which reduced sharply by November. Adherence with face masks was high throughout the semester.

#### Classroom Density

**Figure 4.**
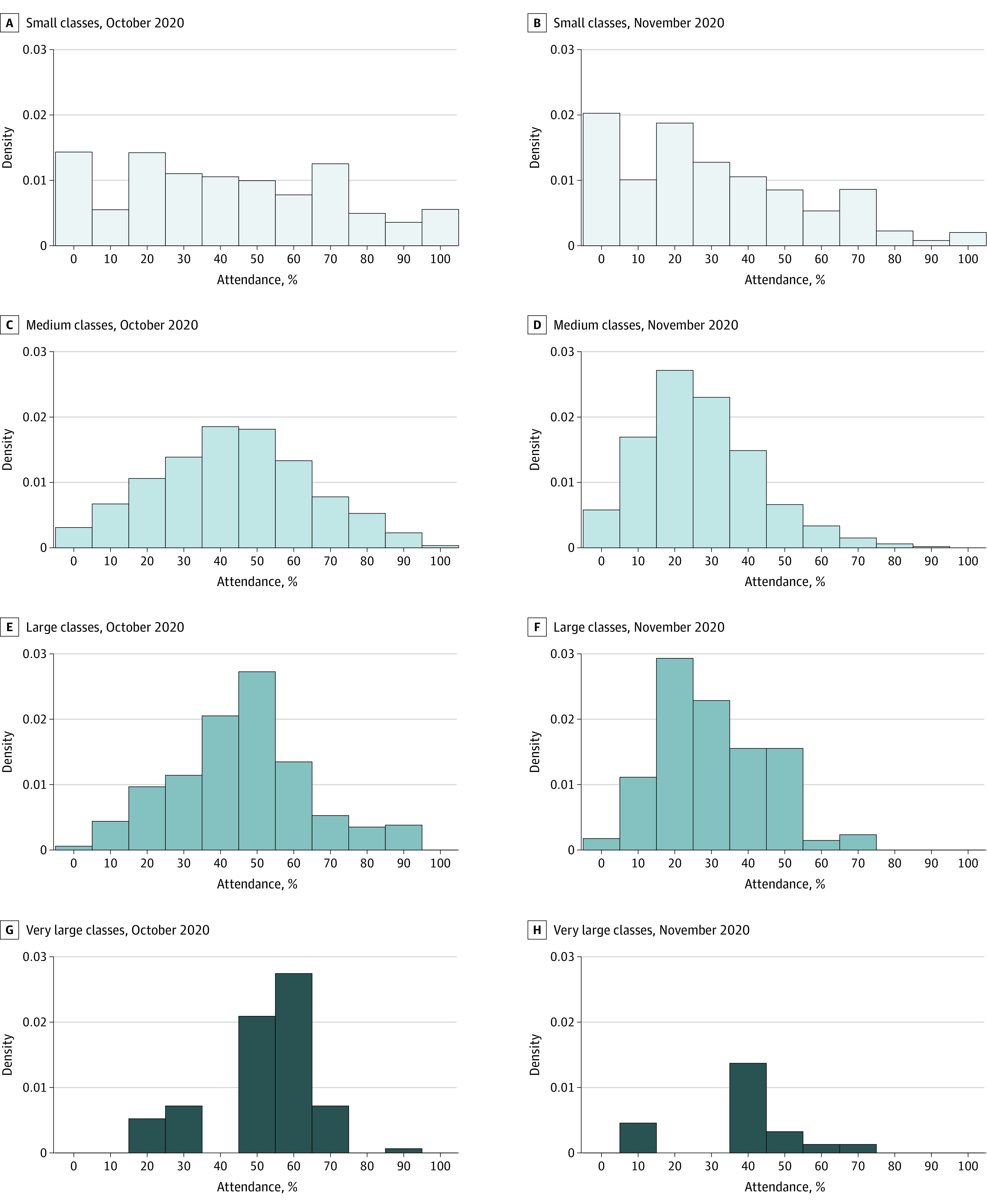
Statistics on Class Attendance for October and November

Class attendance data were not collected systematically. An upper bound on attendance levels is given by the percentage of registered students indicating through Learn from Anywhere that they would attend in person. For medium, large, and very large classes, this number was approximately 45% in October (with medians similar) but decreased to approximately 25% to 30% in November. For small classes, while the mean was similar to the other groups, the variability was substantially greater (Figure 4).

### Epidemiology of COVID-19 at BU and in Boston

#### Changing Epidemiology of COVID-19 in Boston

After statewide control measures were enacted in spring 2020, the mean (SD) number of cases in Suffolk County (which includes Boston) was 0.098 (0.018) cases per 1000 population per day through mid-October.^12^ As state restrictions eased and the weather became colder (and people spent more time indoors), the mean (SD) number of cases increased to 0.300 (0.061) per 1000 population per day by early November, peaking at 0.642 (0.132) per 1000 population per day after Thanksgiving gatherings (Figure 2C). These increasing trends were observed statewide and also in the collection of SARS-CoV-2 from Boston area wastewater, indicating that they were not just due to increased testing in the Boston area.^13,14^

#### COVID-19 Burden and Patterns at BU

During the fall semester, 719 individuals had PCR test results positive for SARS-CoV-2 in the BU community, including 496 students (69.0%), 11 faculty members (1.5%), and 212 staff members (29.5%) (Table). Case numbers increased after holidays, particularly Thanksgiving, and concentrated among undergraduate students and nonfaculty staff. While the incidence rate among students and staff tracked that of Suffolk County (Figure 2C), there were 2 distinctions. First, BU surveillance testing detected many asymptomatic individuals (271 individuals, or 37.7% of our total cases), whereas testing elsewhere in Massachusetts was passive and driven by emergent symptoms. Second, particularly after Thanksgiving, the BU increase in cases preceded that of Suffolk County. This is most likely attributable to BU’s rigorous testing regime.

**Table.** Number of Boston University Individuals With Test Results Positive for SARS-CoV-2 During the Fall 2020 Semester

Characteristic	No. (%)				
	Total	Undergraduate students	Graduate students	Staff	Faculty
Total No. (%)	719 (100.0)	315 (43.8)	181 (25.2)	212 (29.5)	11 (1.5)
Asymptomatic^a^	271 (37.7)	113 (35.9)	51 (28.2)	104 (49.1)	3 (27.3)
Exposure					
Known exposure	370 (51.5)	183 (58.1)	98 (54.1)	84 (39.6)	5 (45.5)
BU exposure (any)	164 (44.3)	108 (59.0)	39 (39.8)	17 (20.2)	0
HHCC	6 (0.8)	3 (1.0)	2 (1.1)	1 (0.5)	0
CC	158 (22.0)	105 (33.3)	37 (20.4)	16 (7.6)	0
Non-BU exposure (any)	206 (55.7)	75 (41.0)	59 (60.2)	67 (79.8)	5 (100.0)
Non-BU HHCC	62 (8.6)	13 (4.1)	15 (8.3)	33 (15.6)	1 (9.1)
Non-BU CC	84 (11.7)	31 (9.8)	28 (15.5)	22 (10.4)	3 (27.3)
Outside BU exposure^b^	44 (6.1)	22 (7.0)	13 (7.2)	8 (3.8)	1 (9.1)
Travel^c^	16 (2.2)	9 (2.9)	3 (1.7)	4 (1.9)	0
Unknown	349 (48.5)	132 (41.9)	83 (45.9)	128 (60.4)	6 (54.6)

Abbreviations: BU, Boston University; CC, close contact; HHCC, household close contact.

^a^ Two individuals refused to provide data on symptoms, reducing sample side to 717 individuals for the asymptomatic variable.

^b^ Outside BU exposure includes individuals who had not been on campus and had no known close contact or recent engagement on campus or with BU community members.

^c^ Travel indicates case returned from out-of-state travel via public transportation (eg, plane, bus, train) 48 hours prior to symptom onset or positive test.

Of 837 students who were close contacts and entered quarantine, 86 (10.3%) had test results positive for SARS-CoV-2 while in quarantine. The test positivity rate for those who self-attested having symptoms was notably higher (174 of 3543 tests [4.9%]) than that of asymptomatic individuals (476 of 462 818 tests [0.1%]), indicating the utility of this feature.

#### Sources and Locations of Transmission

BU contact tracers identified a transmission source for 370 individuals (51.5%), with 206 individuals (55.7%) identifying a source outside BU (Table). Among 5 infected faculty members and 84 infected staff members with a known source of infection, most reported a transmission source outside of BU (all 5 faculty members [100%] and 67 staff members [79.8%]). Students identified more BU contacts as infection sources (39 of 98 graduate students [39.8%] and 108 of 183 undergraduate students [59.2%]) (Table). Notably, BU household contacts were identified as a source of infection less than 1% of the time (Table), and, anecdotally, there were no sustained transmission events in on-campus housing, indicative of the efficacy of efforts to control spread in campus housing. When asked to identify close contacts, students rarely identified classmates. Instead, close contacts tended to be friends and, after Thanksgiving, family (Figure 2D). This indicates that while BU students tended to be more likely to identify another BU affiliate as a source of infection, the contacts leading to infection occurred in places out of reach of BU’s interventions.

## Discussion

This case series from a large university found that strong central leadership; internal communication to students, staff, and faculty; frequent and adaptive testing of students^15,16^; short testing turnaround time; highly effective contact tracing coupled with isolation and quarantine; and vigorous enforcement combined to prevent widespread campus outbreaks of COVID-19 despite the worsening local situation. Although many US higher education institutions reopened in fall 2020, some experienced large COVID-19 outbreaks, forcing a return to online-only education.^17,18,19^ The BU experience is important because BU has an urban campus in a community that experienced high and increasing COVID-19 incidence from August to December 2020, with no option for the university to isolate from the wider community. Despite this, BU benefited from substantial resources, including funding to establish and run a SARS-CoV-2 testing laboratory and sampling centers, a hybrid learning approach (Learn from Anywhere), and diverse expertise among university employees in medical epidemiology, modeling, biostatistics, and public health control measures.

A strong leadership structure with multiple subcommittees targeting important aspects of the response supported the interventions. Frequent communication and coordination between these groups ensured that, if a cluster of cases was emerging, all parties were aware. Thus, testing cadences could be adapted, adherence efforts modified, and messaging adapted to blunt any outbreak risk. This coordinated effort was key to ensuring a high level of adherence and the success of planned interventions.

Short turnaround time of results followed by rapid isolation of individuals who were infected, contact tracing, and quarantine of close contacts were associated with limited transmission in the BU community. Faculty and staff were almost always infected outside of the university campus. While most students with a known source of infection reported another BU affiliate as their contact, these infectious events appeared to occur outside of BU housing and instructional settings, where interventions were targeted. When case clusters appeared in settings the university could not directly target, eg, social or other off-campus gatherings, these were quickly controlled, owing to our intensive testing regimen, rapid contact tracing, and strict enforcement measures. Consequently, no major outbreaks were observed, and the resulting numbers of cases throughout the semester were consistent with our goal of maintaining a linear, rather than exponential, increase in case numbers, which was manageable with our intervention strategies. This also resulted in a relatively low level of use of the available quarantine and isolation rooms during the semester despite concerns during planning for the semester that these could have been quickly overwhelmed if there had been a large outbreak on campus.

Surveillance testing facilitated identification and isolation of many close contacts ahead of contact tracing efforts. Importantly, owing to the surveillance testing system, BU tended to detect increases in cases before the surrounding community, where people were mostly only tested once symptomatic. This is supported by the fact that 37.7% of individuals with positive test results were asymptomatic at the time of isolation, indicating that BU was detecting infections early in the disease course. This finding contradicts recently published research suggesting that BU cases led to infections in the surrounding area in a causal manner.^20^ Limitations to the latter study include the inclusion criteria resulting in only 1 Boston-area institution in the analysis and assuming that community transmission arose from the university campus rather than the converse.

BU’s success is consistent with the current understanding of best COVID-19 control practices. These strategies, aggressive testing, contact tracing, and quarantine and isolation, have been successfully implemented in many countries, including Singapore, South Korea, and Taiwan.^21,22,23^ However, unlike these countries, the BU setting did not allow restriction of travel between the campus and nearby community, making this a strong demonstration of the utility of these approaches despite substantial importation of cases from the surrounding community. This could potentially serve as a model for other institutions nested within a broader community.

BU’s approach carries a high financial cost^24^; BU had to implement budget adjustments, including hiring freezes, salary freezes, and several other cost-cutting measures, to meet the cost of these supplemental services and respond to declining revenue because of pandemic-related changes to operations. Ultimately, the university was able to meet its financial obligations and avoid large layoffs or other consequential financial impacts. Also, providing students an in-person option was beneficial for varying learning styles and meeting immigration requirements. The remote option also benefitted students who could not attend in person owing to health considerations or travel restrictions.

### Limitations

This study has some limitations. Sometimes the contact tracing team was unable to identify contacts owing to students’ reluctance to divulge information regarding where they had been or whom they had been with. In these cases, coordination between the dean of students and the contact tracing team was critical in identifying other students who were associated with the infected student(s) through team or club membership, so an increased frequency of testing (adaptive testing) could be performed.

## Conclusions

In this case series, a multipronged response was associated with controlling SARS-CoV-2 spread on an urban university campus, despite the rising community burden of disease. BU benefited from being a large research university with much of the required expertise for our strategy available within the university, saving money and facilitating substantial control over the operations. This is clearly not feasible for all higher education institutions. This implies that broader efforts in the community, supported by government public health agencies, are required to control spread.

## Notes

Supplement. **eAppendix.** Program Overview **eFigure 1.** Organogram of Boston University’s COVID-19 Recovery Organization **eFigure 2.** Modeling Results Indicating the Relative Impact of Layering Interventions **eFigure 3.** Use of Quarantine and Isolation Housing eReferences
